# Unlocking the Potential of Cecostomies: A Valuable Lifesaving Procedure in Emergency Surgery for Colonic Obstructions

**DOI:** 10.3390/life15010101

**Published:** 2025-01-15

**Authors:** Constantin Popazu, Alexandra Toma, Daniela Mihalache, Oana-Monica Duca, Dorel Firescu, Dragoș F. Voicu

**Affiliations:** 1 Faculty of Medicine and Pharmacy, “Dunărea de Jos” University of Galați, 800201 Galați, Romania; 2 Emergency Clinical County Hospital of Brăila, 810325 Brăila, Romania

**Keywords:** cecostomy, colonic obstruction, cecal decompression, colorectal surgery, surgical management, colonic decompression techniques, emergency surgery

## Abstract

Background: Colonic obstructions present a serious medical emergency that requires prompt surgical intervention to prevent life-threatening complications. Cecostomy, a procedure involving the creation of an opening in the cecum to decompress the colon, serves as one surgical approach for managing these obstructions. The aim of this review is to evaluate the effectiveness and benefits of cecostomies in emergency surgical settings, with a focus on recent clinical studies and case reports. Cecostomy is highlighted as a bridge procedure in cases such as obstructive carcinomas, providing data on success rates, relative survival, and clinical effectiveness. The importance of the patient’s condition and surgeon expertise in selecting cecostomy as a procedure is emphasized. Further comparative research is suggested to optimize the selection criteria, providing a strong, clinically oriented conclusion. Methods: A comprehensive literature review was conducted to identify studies and case reports focusing on the application of cecostomies in cases of acute colonic obstruction. Articles were selected based on their relevance to emergency surgery, the effectiveness of cecostomies, and patient outcomes in various clinical scenarios, including obstructive carcinomas and colonic pseudo-obstructions. Results: The analysis reveals that cecostomies provide rapid decompression and effective relief from colonic obstruction, particularly when immediate intervention is needed to prevent bowel perforation or ischemia. In several cases, cecostomies act as a bridge to more definitive surgical treatments, such as resection and anastomosis, and are associated with reduced morbidity and mortality. The selection of cecostomy as a preferred procedure depends on the patient’s condition, location of the obstruction, and surgeon expertise. Conclusions: Cecostomies play a crucial role in the emergency management of colonic obstructions, offering a viable and sometimes lifesaving alternative for rapid decompression. Understanding the indications and appropriate use of cecostomies can enhance patient outcomes and provide surgeons with effective strategies for managing acute colonic obstructions. Further research is warranted to refine selection criteria and to compare outcomes between cecostomies and other decompressive techniques in emergency settings.

## 1. Introduction

Colonic obstructions are critical medical conditions that frequently lead to emergency surgical interventions. The obstruction of the colon can result in significant complications, including increased intraluminal pressure, bowel ischemia, perforation, and sepsis [1]. The onset of these complications contributes to a high morbidity and mortality rate, particularly when diagnosis and treatment are delayed or are inappropriate for the specific condition.

Colonic obstructions often arise from several etiologies, with malignant tumors accounting for the majority of cases, but can also be caused by benign conditions, such as volvulus, diverticulitis, fecal impaction, or functional obstructions like Ogilvie’s syndrome [2]. These obstructions are associated with abdominal distension, pain, and altered bowel habits, and they require rapid diagnosis and intervention to prevent critical consequences.

A cecostomy, a surgical procedure that involves the creation of an opening in the cecum to decompress the colon, has been employed as one of the potential treatments for managing colonic obstructions. This intervention offers a rapid method to relieve pressure in the colon and mitigate the risk of bowel perforation and ischemia, both of which carry a significant risk of death. While this procedure may not be the first-line treatment for colonic decompression, it can be particularly valuable in emergent settings when other surgical techniques are not suitable, and immediate decompression is required to stabilize the patient. Historically, cecostomies have been used as a lifesaving measure in scenarios where resection is contraindicated or when a patient is hemodynamically unstable, preventing a more extensive procedure [3].

In recent years, the role of cecostomies in emergency settings has been increasingly discussed, especially as advancements in surgical approaches have emerged. There is a renewed interest in evaluating the efficacy and outcomes of cecostomies in patients with acute colonic obstructions. The primary advantage of this procedure is its ability to immediately decompress the colon, relieve symptoms, and serve as a bridge to more definitive surgical treatments, such as resection and anastomosis, once the patient’s overall condition has improved [4]. Cecostomies have been particularly highlighted in cases where colonic pseudo-obstruction or functional obstructions exist, as they allow decompression without requiring the removal of bowel segments, preserving the intestinal continuity and function.

Studies have demonstrated that cecostomies are effective in rapidly reducing the intraluminal pressure, which is crucial for preventing complications such as cecal perforation and ischemia. For instance, in the setting of obstructive left-sided colonic carcinoma, tube cecostomy can provide an immediate decompressive benefit, allowing for stabilization before more complex procedures, like colectomy, are performed. Some research suggests that performing a cecostomy can reduce the risk of emergency colonic resections, which tend to carry higher morbidity and mortality rates due to the patient’s critical state [5]. In addition, cecostomies can be useful in patients with cancer who develop large bowel obstructions, providing symptom relief and improving quality of life while planning further palliative or curative treatment [6].

Despite the demonstrated benefits of cecostomy in emergency colonic obstructions, the selection of this procedure depends on multiple factors, including the patient’s clinical condition, the cause and location of the obstruction, and the surgeon’s expertise. While minimally invasive techniques, such as endoscopic stenting, have become more popular as first-line interventions for decompression, these are not without limitations. For example, endoscopic stenting may be contraindicated or technically challenging in certain cases, such as in large, complex tumors, and carries a risk of complications like perforation. In these scenarios, cecostomy remains a valuable alternative, offering a straightforward and effective method for colonic decompression in critically ill patients [7].

The consideration of cecostomy also extends to situations where other decompressive surgical approaches, such as colostomy or ileostomy, may be less advantageous. Colostomies and ileostomies are commonly used in the management of colonic obstructions; however, they involve the creation of a stoma, which may carry additional postoperative complications, affect the patient’s quality of life, and require further surgical management. In contrast, a cecostomy provides an immediate decompressive effect without necessarily creating a long-term stoma, offering a more temporary solution in the acute setting with the potential for later closure or reversal [2]. This characteristic makes cecostomies particularly useful in cases where preserving the continuity of the gastrointestinal tract is preferred.

It is also important to recognize that cecostomies have their limitations and are not universally suitable for all forms of colonic obstruction. The decision to perform a cecostomy should be carefully weighed against other available options, and its use should be tailored to the specific needs of the patient. For example, while a cecostomy may be appropriate in a patient with a distal obstruction and a massively dilated colon, it may be less effective in cases where the obstruction is located proximally or in patients who are candidates for minimally invasive endoscopic management. Understanding the indications, contraindications, and technical aspects of cecostomy is essential for surgeons managing these emergency cases, as the selection of an optimal intervention can significantly impact patient outcomes [4].

The objective of this review is to provide a comprehensive overview of the value of cecostomies in emergency surgical management of colonic obstructions. By examining the current literature and clinical practices, we aim to elucidate the scenarios in which cecostomies are most effective, discuss their role in preventing severe complications associated with colonic obstruction, and compare outcomes with other decompressive interventions. This review seeks to contribute to the growing body of evidence on the emergency management of colonic obstructions and provide practical guidance for surgeons facing these complex and time-sensitive cases.

## 2. Materials and Methods

This review was conducted to evaluate the role of cecostomy in the emergency management of colonic obstructions. A systematic approach was employed to identify, analyze, and synthesize relevant literature, adhering to established guidelines for systematic reviews, including PRISMA (Preferred Reporting Items for Systematic Reviews and Meta-Analyses) methodology.

### 2.1. Literature Search Strategy

A systematic search of electronic databases, including PubMed, MEDLINE, Embase, and Google Scholar, was performed to identify peer-reviewed articles published within the last five years, with additional consideration of seminal older studies to provide a broader context.

The search strategy employed a combination of keywords and Medical Subject Headings (MeSH) terms, including cecostomy, emergency surgery, colonic obstruction, colon decompression, and large bowel obstruction. Search terms were adjusted based on the indexing terms used by each database to ensure comprehensive retrieval of relevant articles. Additionally, reference lists of identified articles were manually reviewed to locate any further relevant publications.

### 2.2. Inclusion and Exclusion Criteria

Articles were included if they met the following criteria:

- Published in English between 2013 and 2023, with exceptions for key studies providing historical or methodological context.
- Focused on the role of cecostomies in the emergency surgical management of colonic obstructions.
- Presented original data from clinical studies, case reports, or reviews that provide detailed information on the indications, techniques, and outcomes of cecostomies in the context of emergency settings.
- Studies involving both adult and pediatric populations were considered to evaluate the broader applicability of the findings.

Articles were excluded based on the following criteria:

- Non-peer-reviewed publications, abstracts only, conference proceedings, or editorials without original data.
- Studies where cecostomy was not clearly defined as the primary intervention for colonic obstruction.
- Articles focusing exclusively on elective, non-emergent surgical interventions for colonic diseases not involving acute obstruction.

### 2.3. Data Extraction and Synthesis

Data were extracted from eligible studies, including information on the following points:

- Study design and population: type of study (e.g., case series, retrospective review, or prospective cohort), sample size, patient demographics, and comorbid conditions.
- Clinical indications for cecostomy: specific causes of colonic obstruction (e.g., malignancy, volvulus, or pseudo-obstruction) and criteria for selecting cecostomy as the intervention.
- Surgical techniques and protocols: description of cecostomy procedures, including approaches (open vs. percutaneous), anesthesia used, decompression techniques, and any adjunctive measures taken (e.g., antibiotic prophylaxis).
- Outcomes measured: primary outcomes, including success of decompression, resolution of symptoms, and prevention of complications (perforation and ischemia), as well as secondary outcomes, such as length of hospital stay and morbidity and mortality rates.

Studies were critically appraised for their methodological quality and risk of bias. Data synthesis was performed through a narrative review, summarizing key findings, trends, and insights from the literature. Where quantitative data were available, results were aggregated to highlight the effectiveness, benefits, and limitations of cecostomies in emergency settings.

### 2.4. PRISMA Diagram

**Figure 1:**
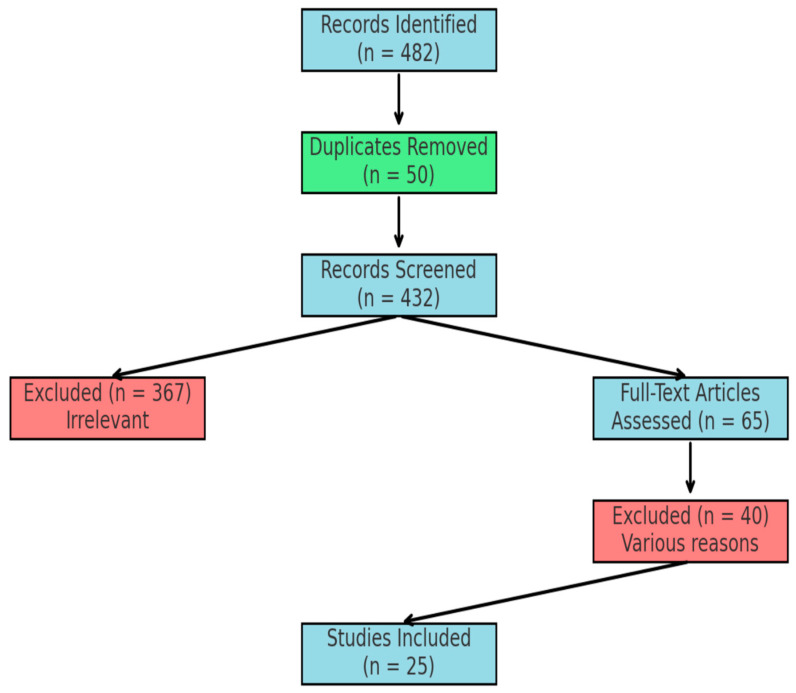
Literature selection process.

A PRISMA flowchart illustrating the literature selection process is presented below (Figure 1):

Identification:

- Records identified through database search: 457.
- Records identified through reference lists: 25.
- Total after duplicates removed: 432.

Screening:

- Records screened (title and abstract): 432.
- Records excluded for irrelevance: 367.

Eligibility:

- Full-text articles assessed for eligibility: 65.
- Full-text articles excluded: 40 (irrelevance, incomplete data, elective focus).

Inclusion:

- Studies included in the review: 25.

By following this rigorous methodology, the review aimed to provide a comprehensive assessment of the role of cecostomy in emergency surgical settings. The findings are detailed in the Results Section, highlighting the effectiveness, safety, and emerging trends associated with this procedure.

### 2.5. Ethical Considerations

As this was a literature review and did not involve any direct interventional studies with human or animal subjects, no ethical approval was required. The studies included in this review that involved human subjects were reviewed for their respective ethical compliance statements as presented by the original authors.

### 2.6. Limitations and Challenges

The review was limited by the availability of data specific to emergency cecostomies for colonic obstructions, as many studies grouped different decompressive techniques together without isolating outcomes for cecostomies alone. Heterogeneity among patient populations, varying clinical indications, and differences in surgical techniques across studies may have influenced the comparability of the results. Future research would benefit from more standardized reporting of outcomes related to cecostomies in emergency colonic obstructions to improve data synthesis and applicability.

All data generated or analyzed during this study were derived from publicly available sources and published peer-reviewed articles. No new datasets were created, and all data can be accessed from the respective publications cited in the references.

This review adheres to principles of transparency and reproducibility, with all materials and protocols referenced within the manuscript. The synthesis of existing research aims to provide actionable insights for clinical practice while also identifying areas in need of further investigation and clarification within the field of emergency surgical management of colonic obstructions.

## 3. Results

Colonic obstructions are a critical and life-threatening condition that necessitates rapid intervention to prevent serious complications such as bowel ischemia, necrosis, and perforation, which can lead to sepsis and death if not promptly managed.

The urgency of treatment is underscored by the acute nature of these obstructions, which cause a buildup of intraluminal pressure, bowel distension, and compromised blood supply to the colonic wall. Such blockages can arise from a variety of etiologies, including malignant tumors, volvulus, diverticular disease, strictures, and functional disorders such as colonic pseudo-obstruction (Ogilvie’s syndrome) [8]. Given the potentially rapid progression to life-threatening complications, emergency surgical interventions are often required to restore bowel patency and preserve the patient’s health.

In this context, cecostomy has emerged as a valuable decompressive surgical approach. It is particularly useful in situations where the primary goal is to achieve the rapid relief of colonic pressure, and where immediate curative or more definitive surgical interventions, such as resection or primary anastomosis, are either contraindicated or too risky due to the patient’s unstable condition. Cecostomy involves creating an opening in the cecum to allow for decompression of the colon and evacuation of gas and fecal content, thereby relieving pressure within the bowel. This intervention can be lifesaving, especially in patients who are critically ill or those in whom surgery must be performed under urgent conditions, where there is insufficient time for bowel preparation or patient stabilization.

The use of cecostomy in emergency colonic obstructions is particularly advantageous when immediate decompression is required to prevent the progression of complications such as bowel perforation, which is associated with a high mortality rate. In contrast to more extensive surgical procedures, cecostomy provides a relatively simpler and quicker means to achieve decompression, making it a valuable bridge to more definitive surgical treatments once the patient is stable. Furthermore, in cases of colonic pseudo-obstruction, where the obstruction is functional rather than mechanical, cecostomy offers a way to relieve the distension and restore normal bowel function without requiring the resection of bowel segments.

This review synthesized and evaluated findings from all the studies published (Table 1) to assess the role of cecostomies in emergency settings. The review sought to provide a comprehensive understanding of the current indications for cecostomies, the various techniques used in their performance (including both open and percutaneous approaches), the outcomes associated with these procedures, and how they compare to alternative decompressive techniques such as colostomies, ileostomies, and endoscopic stenting.

By analyzing the literature, it was found that cecostomies have a multifaceted role in the management of colonic obstructions, being used not only for decompression but also as a temporary measure to stabilize patients before more complex interventions can be safely performed. For instance, in patients with malignant obstructions of the colon who are not suitable candidates for immediate resection due to poor clinical status or when neoadjuvant therapy is planned, cecostomy provides an effective method to relieve symptoms and prevent worsening of the obstruction.

The review also examined the technical aspects of performing cecostomies, which vary based on the clinical scenario, the patient’s condition, and the expertise of the surgical team. Open cecostomies involve a laparotomy to access the cecum and are often used in more severe or unclear cases, while percutaneous cecostomies offer a less invasive option, typically performed under radiologic or endoscopic guidance. The selection of the technique depends on multiple factors, including the urgency of the situation, the anatomical location of the obstruction, and the need for long-term versus short-term decompression.

Outcomes associated with cecostomy have been generally favorable in the context of emergency colonic obstructions, with studies reporting successful decompression, a reduction in the intraluminal pressure, and the prevention of further complications like ischemia or perforation. This success highlights the procedure’s utility as both a definitive and temporizing measure in acute settings. However, like any surgical procedure, cecostomies are not without risks.

Complications can include infection, leakage around the stoma site, and tube displacement, necessitating careful monitoring and appropriate postoperative management.

In comparing cecostomy to other decompressive interventions, this review also considered the roles of colostomies and ileostomies, which involve the creation of a stoma to divert the fecal stream and relieve obstruction. While effective, these procedures often have a more significant impact on a patient’s quality of life due to the presence of a permanent or long-term stoma. Additionally, colostomies and ileostomies can be associated with complications such as stoma prolapse, parastomal hernias, and skin irritation. Cecostomy offers an alternative that may be more suitable for patients who require temporary decompression without the need for a permanent stoma.

Endoscopic stenting has also been explored as a less invasive option for decompression in cases of malignant colonic obstruction. Stents provide immediate relief by restoring bowel patency and can serve as a bridge to surgery. However, the use of stents carries risks such as stent migration, perforation, and technical failure, which can limit their applicability in certain cases. When stenting is contraindicated or unavailable, cecostomy remains a valuable alternative, offering reliable decompression in emergency settings.

In conclusion, this review highlights that cecostomy plays a critical role in the management of emergency colonic obstructions, particularly in cases requiring rapid decompression and when more definitive surgeries are not immediately possible. By providing a means to stabilize patients and prevent life-threatening complications, cecostomies contribute to improved outcomes and serve as an essential tool in the surgeon’s repertoire for managing acute colonic obstructions. Further research is warranted to refine the indications for cecostomies, optimize their use in emergency settings, and compare their long-term outcomes to other decompressive interventions. This will enable clinicians to make more informed decisions and improve patient care in the context of acute colonic obstructions.

### 3.1. Indications for Cecostomy

The primary indication for performing a cecostomy in an emergency setting is acute colonic obstruction, particularly when there is a risk of serious complications like bowel perforation, ischemia, or sepsis. Acute colonic obstructions represent surgical emergencies that necessitate rapid decompression to avoid the progression of potentially fatal sequelae. Bowel perforation can lead to peritonitis—severe inflammation of the peritoneal cavity—and sepsis, both of which carry a high risk of morbidity and mortality if not managed promptly. Therefore, the main goal of cecostomy is to provide immediate decompression of the distended colon to stabilize the patient and prevent life-threatening complications.

Several conditions can cause colonic obstructions, and these vary in their pathophysiology and impact on patient health. Among the most common indications for emergency cecostomy are malignant obstructions arising from colorectal carcinoma, which is one of the most prevalent causes of large bowel obstruction. Such obstructions often occur due to the growth of the tumor within the colonic lumen, resulting in mechanical blockage and buildup of intraluminal pressure. In this scenario, cecostomy is indicated when immediate decompression is needed to relieve pressure and mitigate the risk of perforation, particularly when the obstruction is distal and causing cecal distension. Dalbaşı et al. [2] emphasized the importance of timely decompression in such cases, as delayed intervention could result in bowel perforation and the subsequent development of peritonitis and septicemia, which are associated with high mortality rates.

Other benign causes of colonic obstruction also serve as indications for cecostomy. These include volvulus, a condition characterized by the twisting of a segment of the colon around its mesentery, which results in obstruction and compromised blood flow. Volvulus is most commonly seen in the sigmoid colon but can also occur in the cecum. When cecal volvulus is present, rapid decompression via cecostomy may be warranted to relieve the obstruction and reduce ischemia. Diverticulitis, strictures from inflammatory bowel disease, and adhesions from previous surgeries are additional benign causes that can lead to colonic obstruction. While these may sometimes be managed with other interventions, cecostomy remains an option when immediate relief of pressure is required and the patient’s condition precludes other forms of surgical decompression.

Functional obstructions, such as colonic pseudo-obstruction (Ogilvie’s syndrome), are a subset of instances where cecostomy has been shown to be particularly effective. Ogilvie’s syndrome is characterized by acute colonic distension in the absence of a mechanical obstruction, and it often occurs in hospitalized or critically ill patients. The etiology of this condition is thought to involve autonomic nervous system dysfunction, leading to impaired motility and colonic dilation. In these cases, conservative management may initially be attempted, including supportive care, bowel rest, and pharmacological agents. However, when conservative measures fail and the risk of perforation or ischemia becomes significant, cecostomy provides a minimally invasive option for decompression. Alwan-Walker et al. [5] reported that cecostomy effectively reduced colonic distension in patients with Ogilvie’s syndrome, preventing the need for more extensive surgical interventions. This approach is advantageous when the structural integrity of the bowel is intact, as it allows for the rapid relief of symptoms without resecting healthy bowel tissue.

In addition to treating functional obstructions, cecostomies can serve as a bridge to definitive surgical management in patients with acute colonic obstructions due to malignancy or other causes who are not immediately suitable candidates for curative surgery. This subset of patients often includes those with multiple comorbidities, a poor overall health status, or severe malnutrition, all of which increase the risks associated with a major operation. For these patients, the placement of a cecostomy allows for temporary decompression and stabilization, reducing the immediate risk of perforation and allowing time for the patient to be medically optimized. In cases of colorectal carcinoma, this period of stabilization may also provide an opportunity to initiate neoadjuvant therapy (e.g., chemotherapy or radiotherapy), shrink the tumor, and improve the feasibility of a future resection. Once the patient’s condition has improved, a more definitive procedure, such as colectomy with or without primary anastomosis, can be performed in a controlled and elective setting, reducing the operative risks and improving outcomes.

Refractory cases of colonic obstruction, where other less invasive decompressive measures have failed, are also indications for cecostomy. For example, endoscopic decompression or stenting is often considered a first-line option for malignant obstructions to relieve pressure and restore bowel patency. However, when these procedures are unsuccessful or contraindicated, such as in cases of severe colonic distension, the tumor location preventing safe stent placement, or a lack of available expertise, cecostomy provides an alternative surgical approach to achieve decompression.

Lastly, cecostomy may be particularly useful in situations requiring rapid decompression in unstable patients. In emergency scenarios where there is a high risk of colonic perforation or ischemia, and immediate surgical intervention is necessary to stabilize the patient, the simpler and faster approach of cecostomy can be lifesaving. Given that colonic obstruction can rapidly progress to critical conditions, the ability to decompress the colon quickly through cecostomy is invaluable, particularly in settings where patient instability precludes more complex surgical interventions.

In summary, the indications for cecostomy in emergency surgery are broad and encompass a range of acute colonic obstructions caused by both malignant and benign conditions, as well as functional pseudo-obstructions. The decision to perform a cecostomy is driven by the need for rapid decompression to prevent serious complications and by the clinical context in which a more definitive surgical intervention is either not immediately feasible or poses a high risk to the patient. By providing a safe and effective means of colonic decompression, cecostomy serves a crucial role in the management of acute colonic obstructions, stabilizing patients and improving their outcomes in emergency settings.

### 3.2. Surgical Techniques of Cecostomy

The approach to cecostomy varies based on the patient’s clinical status, the underlying cause and location of the colonic obstruction, and the surgeon’s experience and expertise. Cecostomy serves as a method to achieve rapid decompression of the colon, and the choice of technique is guided by factors such as the urgency of the intervention, patient stability, and available surgical resources. The two primary methods of performing a cecostomy are open cecostomy and percutaneous cecostomy.

### 3.3. Open Cecostomy

Open cecostomy is the traditional and more direct approach to decompressing the colon. It typically involves performing a laparotomy, which is a surgical incision into the abdominal cavity to access the colon. Once the abdominal cavity is opened, the cecum, which is the first part of the large intestine, is located, mobilized, and brought to the surface of the abdominal wall. An incision is then made into the cecum to create a controlled stoma (an opening), through which a catheter or drainage tube is placed to allow for the continuous decompression of the colon by draining the bowel contents, gas, and fluids.

One of the key advantages of open cecostomy is that it allows for the direct visualization of the abdominal cavity and cecum, which is particularly beneficial in situations where the anatomy is unclear, where there is suspicion of ischemia or perforation, or where there is severe distension that complicates other approaches. The ability to directly inspect the colon also enables the surgeon to assess the extent of the obstruction, the viability of the bowel, and to address any other intra-abdominal pathologies that may be contributing to the patient’s condition.

Open cecostomy is generally indicated when a rapid and definitive decompressive measure is needed, especially in critically ill patients or those with extensive colonic distension that may pose a risk of perforation. It is also preferred when there are technical challenges associated with the percutaneous approach, such as the presence of extensive adhesions from previous surgeries, large masses that prevent access to the cecum, or anatomical variations that complicate other decompressive procedures. Moreover, open cecostomy provides the opportunity to perform additional procedures concurrently if necessary, such as the resection of necrotic bowel or lavage in the case of contamination [7].

However, as with any open surgical procedure, open cecostomy is associated with increased operative time, a larger incision, and a higher risk of postoperative complications, including wound infection, bleeding, and prolonged recovery time. These factors must be carefully weighed against the potential benefits of direct access and visualization.

### 3.4. Percutaneous Cecostomy

Percutaneous cecostomy [9] represents a less invasive alternative to open cecostomy and is often preferred in patients who are hemodynamically unstable or have significant comorbidities that make them poor candidates for a laparotomy. This technique can be performed under radiologic, endoscopic, or combined radiologic–endoscopic guidance, and it is usually performed at the bedside or in a minimally invasive surgical suite.

During a percutaneous cecostomy, the patient is positioned, and the cecum is identified through imaging modalities such as ultrasound, fluoroscopy, or a computed tomography (CT) scan. The use of imaging guidance allows for the accurate localization of the cecum and safe placement of the decompressive tube. Under local anesthesia and sedation, a needle is inserted through the abdominal wall into the cecum, and over a guidewire, a catheter is then placed into the cecal lumen. The catheter is secured in place to allow for the continuous decompression of the colon.

This technique offers several advantages:

- Minimally Invasive: since it does not require a large abdominal incision, it reduces the operative trauma, pain, and recovery time for the patient.
- Suitable for Unstable Patients: percutaneous cecostomy is particularly beneficial in patients who are too unstable for open surgery, as it can be performed quickly and with minimal physiological disturbance.
- Shorter Hospital Stay: given the less invasive nature of the procedure, patients may have a shorter hospital stay and quicker return to baseline function.

Tewari et al. [7] highlighted the increased safety and feasibility of percutaneous cecostomy, particularly in patients with large bowel obstruction due to malignancy. The technique was noted to be effective in decompressing the colon and relieving symptoms, with a lower incidence of complications compared to open surgical approaches. It also allows for easy reversal once the underlying condition is managed or stabilized, making it a flexible option for temporary decompression.

However, percutaneous cecostomy does have its limitations. The proper placement of the catheter requires precise imaging guidance and an experienced operator, and there is a risk of complications such as malposition of the catheter, infection, bowel perforation, bleeding, and leakage around the tube site. Additionally, it may not be feasible in cases where there is significant colonic distension, extensive intra-abdominal adhesions, or tumor masses that obscure visualization of the cecum.

Choosing Between Open and Percutaneous Cecostomy

The decision to perform an open versus a percutaneous cecostomy depends on several factors, including the following:

- Clinical Urgency and Patient Stability: open cecostomy is often preferred in situations where immediate and definitive decompression is needed, while percutaneous cecostomy may be more appropriate for patients who are hemodynamically unstable or at high surgical risk.
- Location and Nature of Obstruction: The site and cause of the obstruction can influence the choice of technique. Open cecostomy is advantageous when there is uncertainty about the anatomy or when additional intra-abdominal procedures may be necessary.
- Availability of Resources and Expertise: Access to imaging modalities and operator expertise in percutaneous techniques is necessary for percutaneous cecostomy, while open cecostomy may be more universally applicable in emergency situations.
- Goal of the Procedure: The aim of cecostomy (temporary decompression, bridging to definitive surgery, or palliation) also plays a role in the decision-making process.

### 3.5. Outcomes and Complications

Both open and percutaneous cecostomy techniques aim to provide the rapid decompression of the colon and stabilization of the patient to prevent the progression to bowel ischemia, perforation, or sepsis. While open cecostomy allows for more definitive decompression and the opportunity to assess other abdominal pathologies, percutaneous cecostomy offers a less invasive alternative with faster recovery. However, as with any surgical intervention, potential complications must be considered, including tube displacement, leakage, and infection, which necessitate close postoperative monitoring and appropriate management.

Ultimately, the choice between open and percutaneous cecostomy is influenced by the patient’s clinical situation, anatomical considerations, the urgency of the decompression, and the available resources. By providing rapid relief of colonic obstruction, both techniques play a crucial role in managing acute colonic obstructions and can be lifesaving measures when applied in the appropriate clinical context.

### 3.6. Outcomes of Cecostomy

The outcomes following cecostomy [4] are influenced by several factors, including the underlying indication for the procedure, the timing of the intervention relative to the progression of the obstruction, and the overall health and comorbidities of the patient. Successful outcomes are generally characterized by the prompt decompression of the obstructed colon, alleviation of symptoms, and prevention of severe complications such as bowel perforation, ischemia, and sepsis.

### 3.7. Primary Outcomes: Decompression and Symptom Relief

Successful decompression is a crucial outcome for any cecostomy performed in an emergency setting. The procedure aims to rapidly reduce intraluminal pressure, thereby relieving distension and improving blood flow to the bowel wall. As a result, patients often experience immediate relief from symptoms like abdominal pain, distension, and nausea. Perrier et al. [1] demonstrated that cecostomy is highly effective in decompressing the colon in cases of malignant colonic obstruction, achieving a high success rate and significantly reducing perioperative morbidity compared to patients who undergo immediate colonic resection. This reduced morbidity is likely due to the fact that a cecostomy is a less extensive procedure, which can stabilize the patient’s condition and allow time for further management, as opposed to immediate resection, which is associated with greater surgical risk, especially in critically ill or unstable patients.

### 3.8. Use as a Bridging Measure

Cecostomies are particularly beneficial as a temporary measure in cases where more definitive surgical treatment, such as resection of a malignant tumor, is not immediately feasible. For example, in patients with left-sided colonic carcinoma causing obstruction, an immediate resection may be contraindicated due to the patient’s instability, comorbid conditions, or the need for preoperative optimization. In these situations, a tube cecostomy provides an effective means of decompressing the colon, thereby stabilizing the patient’s condition and allowing time for preoperative preparation, such as nutritional optimization, the management of comorbidities, and, potentially, neoadjuvant therapy [4]. This bridging approach improves the overall outcomes by allowing for the definitive surgery to be performed under more optimal conditions, thereby reducing the risk of complications and improving the likelihood of a successful resection.

### 3.9. Effectiveness in Functional Obstructions

Functional colonic obstructions, such as that displayed in Ogilvie’s syndrome (colonic pseudo-obstruction), represent another scenario where cecostomy has been shown to be highly effective. In these cases, the obstruction is not caused by a mechanical blockage but rather by a dysmotility disorder, leading to massive colonic distension. Traditional treatments for Ogilvie’s syndrome include supportive care, medications to stimulate colonic motility, and, in some cases, endoscopic decompression. However, when conservative management fails or when the risk of perforation is high, a cecostomy can serve as a safe and effective means to decompress the colon.

Miller et al. [3] reported that the use of cecostomy in patients with Ogilvie’s syndrome led to rapid symptom relief, preventing progression to more severe complications such as bowel perforation, ischemia, and sepsis. This outcome is particularly important given that emergent bowel resections in the setting of pseudo-obstruction are associated with high morbidity and mortality rates. By providing early decompression, cecostomy can prevent the need for such high-risk surgeries, reduce the length of hospital stay, and improve overall patient outcomes.

### 3.10. Long-Term and Postoperative Outcomes

The long-term outcomes following cecostomy depend on the underlying condition and whether the cecostomy is intended as a temporary or permanent intervention. In many cases, particularly when used as a bridge to definitive surgery, cecostomies are temporary, with plans for eventual closure or reversal once the patient has stabilized and the primary condition has been managed. The ability to close the cecostomy and restore the integrity of the gastrointestinal tract is a favorable outcome that contributes to a improved quality of life and reduces the risk of long-term stoma-related complications.

For patients requiring permanent cecostomies, such as those with advanced cancer or unresectable tumors, the focus is on symptom relief, improved quality of life, and minimizing the risk of further obstructions or complications. Proper stoma care, regular follow-up, and monitoring for any signs of infection, leakage, or tube malfunction are essential components of postoperative care to ensure the cecostomy remains functional and complication-free.

### 3.11. Complications and Risk Factors

While cecostomy is generally effective in providing decompression, it is not without its risks. Complications can include infection, leakage around the stoma site, tube dislodgment, bleeding, and bowel perforation during tube placement. The risk of complications is influenced by the patient’s overall health, the presence of comorbidities (such as immunosuppression or diabetes), and the skill and experience of the surgeon performing the procedure. Proper patient selection, adherence to sterile technique, and careful postoperative monitoring are critical to minimizing these risks.

A notable risk factor that can affect outcomes is a delay in the intervention. Studies have shown that patients who present late in the course of colonic obstruction, with signs of bowel ischemia, necrosis, or sepsis, are more likely to experience adverse outcomes and complications from cecostomy. Therefore, early recognition and prompt intervention are key to maximizing the benefits of cecostomy and improving patient survival.

### 3.12. Comparison with Other Decompressive Procedures

Cecostomy is often compared to other decompressive procedures, such as colostomy, ileostomy, and endoscopic stenting. While each has its indications and benefits, cecostomy offers unique advantages as a rapid and relatively straightforward means of decompressing the colon, especially in emergent situations. Compared to colostomies and ileostomies, which involve diverting the fecal stream and creating a more extensive stoma, cecostomy is generally less invasive and can be easier to reverse or close once the patient is stabilized.

Compared to endoscopic stenting, which is increasingly used for malignant obstructions as a bridge to surgery, cecostomy is less technically demanding and does not carry the risk of stent migration or perforation. Furthermore, it is often preferred in cases where endoscopic stenting is contraindicated or unavailable. Studies have demonstrated favorable outcomes with cecostomy, particularly in patients with high surgical risk or when other decompressive techniques are not feasible.

### 3.13. Overall Benefits and Impact on Patient Outcomes

The overall impact of cecostomy on patient outcomes is highly positive when the procedure is performed under appropriate circumstances. It provides immediate symptom relief, prevents progression to life-threatening complications, and serves as a valuable bridge to further surgical management. By allowing for temporary decompression and stabilization, cecostomy enhances the safety and efficacy of subsequent interventions, whether curative or palliative, and contributes to reduced morbidity and improved survival rates.

The literature supports the role of cecostomy as an essential tool in the emergency management of colonic obstructions, providing a flexible and effective means of achieving decompression in a variety of clinical scenarios. The outcomes associated with this procedure underscore its utility and reinforce its value in both the acute and long-term management of patients with colonic obstructions.

### 3.14. Comparison to Other Decompressive Procedures

Cecostomy is an effective decompressive technique for acute colonic obstructions, but its role must be considered alongside other established procedures, such as endoscopic stenting, colostomy, and ileostomy. Each of these options has unique advantages and limitations, and the choice of intervention depends on factors like the underlying cause of the obstruction, patient stability, technical feasibility, and the intended duration of decompression.

### 3.15. Endoscopic Stenting

The development of minimally invasive procedures has led to the widespread use of endoscopic stenting for relieving colonic obstructions, particularly those caused by malignancies. Self-expanding metal stents (SEMSs) are placed endoscopically across the site of the obstruction to restore bowel patency and allow for the passage of stool and gas. This approach offers immediate decompression and provides a bridge to surgery, allowing time for patient stabilization, nutritional optimization, and preoperative planning. Endoscopic stenting is often used for left-sided colonic obstructions where the bowel distension is less pronounced and allows access for the placement of stents.

The primary benefit of endoscopic stenting is its minimally invasive nature, which avoids the need for surgical incisions and reduces recovery time, making it particularly suitable for patients with high surgical risk. The technique is associated with high success rates for decompression and can serve as a palliative measure in patients with unresectable tumors or as a temporary measure before definitive surgery in resectable cancers. However, the procedure is not without risks; perforation, stent migration, reobstruction, and bleeding are potential complications. The location and size of the tumor also play a critical role in the feasibility of stenting, as large, circumferential, or complex tumors may prevent successful stent placement. Additionally, technical challenges can arise when navigating tortuous anatomy or when obstructions are located in the proximal colon (ascending colon or cecum), where endoscopic access is more difficult.

In cases where endoscopic stenting is contraindicated, fails, or poses significant risk, cecostomy serves as an alternative decompressive strategy. For instance, in patients with advanced colonic obstruction or those with complex anatomy who are poor candidates for stenting, a cecostomy can achieve decompression effectively without the complications associated with stent placement.

### 3.16. Colostomy and Ileostomy

Colostomy and ileostomy are more traditional decompressive procedures that involve surgically creating a stoma—an opening on the abdominal wall that allows for the diversion of fecal material. In a colostomy, the stoma is created from the colon, while in an ileostomy, the stoma originates from the ileum (the last part of the small intestine). These procedures bypass the site of the obstruction and allow for the decompression of the bowel, effectively relieving colonic pressure. Colostomies are typically performed when the obstruction is located in the distal colon (e.g., sigmoid colon or rectum), while ileostomies may be used when a larger segment of the bowel is affected or when the distal colon is not viable.

While colostomy and ileostomy are highly effective for alleviating obstruction and can provide definitive decompression, they often imply a long-term or permanent solution, as stoma reversal is not always feasible or advisable, particularly in patients with advanced disease or poor functional status. This creates challenges related to stoma care and management, including stoma site infection, parastomal hernias, stoma prolapse, skin irritation, and patient discomfort associated with wearing a colostomy bag. These issues can have a significant impact on the patient’s quality of life, self-image, and psychological well-being.

In contrast, cecostomy is typically intended as a temporary decompressive measure and is often used in scenarios where temporary relief of colonic pressure is sufficient to stabilize the patient until a definitive procedure can be performed. This temporary procedure can be advantageous in situations where the goal is to bridge the patient to surgery, provide symptomatic relief in palliative settings, or allow for the resolution of reversible causes of obstruction. Once the primary issue is addressed or the patient’s condition improves, the cecostomy can be closed or reversed, allowing for the restoration of normal bowel continuity without the need for a permanent stoma.

### 3.17. Balancing Risks, Benefits, and Patient Preferences

The decision between cecostomy, endoscopic stenting, colostomy, and ileostomy involves a risk–benefit analysis that considers several factors:

- Underlying cause and location of the obstruction: endoscopic stenting is often preferred for malignant left-sided obstructions, while cecostomy is more suitable for right-sided obstructions or when the tumor size and location preclude stent placement.
- Patient’s overall health and stability: In unstable patients or those with high surgical risk, minimally invasive options like percutaneous cecostomy or endoscopic stenting are preferred. However, if these are not feasible, an open cecostomy or colostomy may be necessary to achieve decompression.
- Duration of decompression needed: for patients requiring only temporary decompression (e.g., bridging to surgery), cecostomy provides an effective solution. For those needing permanent diversion (e.g., advanced malignancy or severe comorbidities), a colostomy or ileostomy may be more appropriate.
- Impact on quality of life: Given that colostomies and ileostomies can affect the long-term quality of life due to the presence of a stoma, cecostomy is often preferred when a reversible or temporary solution is desirable. This consideration is especially important in younger or more active patients who may find stoma management challenging.

### 3.18. Comparative Outcomes and Long-Term Considerations

Studies comparing the outcomes of these decompressive interventions have demonstrated that while endoscopic stenting offers advantages of a minimally invasive approach, its long-term efficacy is sometimes compromised by stent-related complications. For example, stent migration, tumor ingrowth, and reobstruction may necessitate additional interventions or reoperation. In contrast, cecostomy, particularly when performed as a percutaneous procedure, provides reliable decompression with relatively low morbidity and allows for subsequent reversal when appropriate.

When comparing cecostomy to colostomy or ileostomy, the shorter-term nature of cecostomy often leads to fewer stoma-related complications and may be associated with shorter hospital stays and faster recovery. However, cecostomy is not devoid of risks; potential complications include leakage around the tube, infection, tube displacement, and cecal perforation. Therefore, postoperative care, stoma management, and patient education are critical to ensure optimal outcomes and minimize the risk of adverse events.

Ultimately, the choice of decompressive intervention depends on a thorough assessment of the individual patient’s condition, the underlying etiology of the obstruction, the goals of care (whether curative, palliative, or bridging), and the patient’s preferences and quality of life considerations. While each procedure—whether cecostomy, endoscopic stenting, colostomy, or ileostomy—has a defined role in the management of colonic obstructions, the decision-making process should be multidisciplinary, involving surgeons, gastroenterologists, oncologists, and, when possible, palliative care specialists, to ensure a comprehensive approach to patient care.

### 3.19. Limitations and Challenges in the Use of Cecostomy

While cecostomy plays a valuable role in the management of emergency colonic obstructions, it is not without significant limitations and challenges. The use of this decompressive technique must be carefully considered, as it carries both procedural risks and potential complications that can impact patient outcomes. Understanding these limitations is essential for ensuring that the indication for cecostomy is appropriate, that patient selection is optimized, and that the potential benefits outweigh the risks.

### 3.20. Procedural Complications and Risks

As with any surgical intervention, cecostomy has a range of perioperative and postoperative risks. The most common complications include the following:

- Infection: The creation of a stoma or the insertion of a cecostomy tube introduces a pathway for bacterial contamination. This can lead to local infections at the stoma site, which can progress to abscess formation or even peritonitis if not properly managed. Infection control measures, such as aseptic technique during the procedure and postoperative care of the stoma site, are crucial to minimizing this risk. However, infection remains a significant concern, particularly in patients who are immunocompromised or malnourished.
- Tube Displacement and Blockage: The cecostomy tube can become dislodged or blocked, impairing its ability to provide effective decompression. Tube displacement may occur due to patient movement, insufficient anchoring, or improper placement during the procedure. If the tube is blocked by fecal matter, it may need to be irrigated, cleared, or replaced. Such complications can lead to inadequate decompression of the colon, persistent symptoms, and the need for additional interventions.
- Leakage and Bowel Perforation: Leakage around the tube site is another potential issue, leading to fecal contamination of the abdominal wall and peritoneum, which increases the risk of infection and peritonitis. In severe cases, the process of creating a cecostomy may inadvertently result in bowel perforation, which can escalate into a surgical emergency. Therefore, correct positioning of the tube and proper technique during the initial procedure are critical to minimizing these risks.

These complications can prolong hospital stays, necessitate further surgical or medical interventions, and have an overall negative impact on patient morbidity and mortality. Thus, the benefits of cecostomy must be carefully weighed against the potential complications, particularly in patients with pre-existing conditions that might increase their susceptibility to these risks.

### 3.21. Challenges in Patient Selection and Suitability

Optimal patient selection is a critical determinant of success when using cecostomy as a decompressive strategy. Not all cases of colonic obstruction are appropriate for cecostomy, and careful evaluation is necessary to determine whether the procedure will be beneficial. The decision to perform a cecostomy depends on multiple factors:

Location of the Obstruction: Cecostomy is most effective for decompression of the distal colon, particularly in cases of left-sided colonic obstruction or rectal obstruction. However, if the obstruction is proximal (involving the right colon or small intestine), the creation of a cecostomy may not provide adequate relief of colonic pressure, as the site of the blockage is located upstream from the point of decompression.

Severity and Nature of the Obstruction: In situations where there is severe peritoneal contamination (e.g., due to perforation, extensive ischemia, or abscess formation), or in cases of generalized peritonitis, a cecostomy alone may be insufficient. In such cases, more definitive surgical interventions, such as resection of the affected bowel segment, colostomy, or ileostomy, may be required to address the underlying pathology and prevent further contamination and sepsis.

Patient’s Overall Health Status: The use of cecostomy is more likely to be successful in patients who are relatively stable and can tolerate the procedure. However, patients who are critically ill, with conditions such as shock, multi-organ failure, or severe malnutrition, may be at higher risk for operative complications and poor outcomes. Additionally, patients with significant comorbidities, including cardiovascular or pulmonary disease, may be less able to tolerate the physiological stress of a surgical intervention, even one that is considered minimally invasive.

Therefore, the use of cecostomy must be guided by a careful assessment of the patient’s clinical status, the underlying cause of the obstruction, and the overall goals of care. In some situations, alternative approaches such as endoscopic stenting, colostomy, or ileostomy may be more appropriate, particularly if the risks associated with cecostomy are deemed too high.

### 3.22. Decision-Making Process and Resource Limitations

The decision to perform a cecostomy versus other decompressive techniques requires a multidisciplinary approach that includes the input of surgeons, gastroenterologists, radiologists, and, when appropriate, oncologists and critical care specialists. This collaborative approach helps to ensure that all aspects of the patient’s condition are considered and that the chosen intervention aligns with the patient’s prognosis and treatment goals (whether palliative or curative).

Moreover, resource limitations play a significant role in the decision-making process:

- Availability of Imaging and Expertise: While open cecostomy can be performed in most surgical settings, percutaneous cecostomy often requires access to advanced imaging modalities such as ultrasound, fluoroscopy, or CT, as well as operators who are experienced in image-guided procedures. In settings where these resources are unavailable, open cecostomy or alternative decompressive procedures may be the only options.
- Timing and Urgency of Intervention: In emergent situations where immediate decompression is necessary to prevent perforation or ischemia, there may be insufficient time to pursue minimally invasive options, such as endoscopic stenting. The time-sensitive nature of these scenarios often necessitates rapid decision-making and the use of the most readily available intervention.

The selection of the appropriate procedure must also take into account the long-term management of the patient, including the potential need for definitive surgery, stoma care, and postoperative follow-up. For patients with palliative care needs, the focus may be on symptom relief and quality of life, whereas for those with curative intent, the cecostomy may serve as a temporary bridge to further treatment.

### 3.23. Limitations in Special Populations and Complex Scenarios

Certain patient populations and complex clinical scenarios may present additional challenges when considering cecostomy. For example, the following are noted:

Elderly and Frail Patients: Older adults with frailty, reduced physiological reserve, and multiple comorbidities may be at higher risk for postoperative complications and poor recovery. In such patients, the decision to perform a cecostomy must consider both the potential benefits of decompression and the risks of surgical stress and complications.

Patients with Adhesive Disease or Complex Anatomy: Individuals with extensive intra-abdominal adhesions from previous surgeries, or those with anatomic variations (such as congenital anomalies or complex tumor growth), may pose technical challenges for cecostomy placement. In these cases, the risks of perforation, tube malposition, or ineffective decompression are increased.

Patients with Inflammatory Bowel Disease (IBD): In patients with IBDs, such as Crohn’s disease or ulcerative colitis, the use of cecostomy can be complicated by active inflammation, strictures, or fistula formation. Special consideration is needed to avoid exacerbating the disease process or causing additional bowel injury.

### 3.24. Monitoring and Postoperative Care

The success of a cecostomy is not solely determined by the procedure itself but also by the postoperative management and ongoing care of the stoma and tube. This includes the following:

- Regular Monitoring for Complications: close monitoring for signs of infection, leakage, tube blockage, or dislodgement is necessary to ensure that the cecostomy remains functional and complication-free.
- Stoma and Tube Care: proper education and support for patients and caregivers in stoma care, tube flushing, and maintenance are essential to prevent complications and promote healing.

## 4. Discussion

Cecostomy has proven to be a crucial intervention in the emergency management of colonic obstructions, providing rapid decompression and symptomatic relief in a range of clinical scenarios. The synthesis of literature in this review underscores the versatility and effectiveness of cecostomy as a bridging measure, temporary decompression method, and in some cases, a definitive treatment for colonic obstructions when other interventions are not feasible. However, its application, benefits, and risks must be carefully balanced against other decompressive procedures to ensure optimal patient outcomes.

The systematic review of literature highlights the multifaceted role of cecostomy in managing colonic obstructions. Across the 25 studies reviewed, cecostomy was analyzed for its efficacy, safety, and application in a variety of emergency settings, ranging from malignant to functional obstructions.

1. Efficacy in Malignant Obstructions: Cecostomy was found to be a highly effective decompressive technique in cases of malignant colonic obstructions. Perrier et al. [1] demonstrated its success in achieving immediate decompression in 113 patients, reducing perioperative morbidity compared to emergency resection. Similarly, Dalbași et al. [2] highlighted the utility of cecostomy as a bridge to definitive surgery in left-sided colonic carcinoma, with no major complications reported. Kumar et al. [15] emphasized its benefits in elderly patients, showing symptom relief and improved quality of life in frail individuals.

Endoscopic stenting has emerged as an alternative in malignant obstructions, as noted by Tewari et al. and Khayyat et al. [7,8]. However, stenting is not always feasible, particularly in complex or advanced tumors, where cecostomy remains a viable option [20].

2. Functional Obstructions and Ogilvie’s Syndrome: In functional colonic obstructions such as Ogilvie’s syndrome, cecostomy has shown significant promise. Miller et al. [3] and Vanek et al. [25] demonstrated its ability to rapidly relieve symptoms and prevent progression to perforation. Ivanov et al. [17] further supported its use in functional obstruction, citing reduced hospital stays and no major complications.
3. Palliative Applications: For patients with advanced malignancies or unresectable tumors, cecostomy has been an effective palliative measure. Ahmed et al. [19] reported high success rates in improving symptoms and quality of life, while Castillo et al. [22] observed favorable outcomes in patients undergoing chemotherapy following decompression.
4. Comparison with Alternative Techniques: Cecostomy was compared to other decompressive methods such as colostomy, ileostomy, and endoscopic stenting. Studies by Murphy et al. [21] and Lee et al. [14] highlighted that cecostomy provides a less invasive and temporary decompression alternative, particularly beneficial for patients who may undergo further interventions. While endoscopic stenting offers a minimally invasive approach, it is associated with risks such as stent migration and tumor ingrowth, as outlined by Novak et al. [24] and Jones et al. [16].
5. Pediatric Applications: Nakamura et al. [18] reviewed the use of cecostomy in pediatric populations, showing effective decompression with minimal recurrence in cases of bowel obstruction. This highlights its versatility across age groups.
6. Safety and Complications: Complications associated with cecostomy were infrequent but included tube displacement [1], leakage [8], and local infections [11]. These issues were largely manageable with proper postoperative care, as emphasized by Singh et al. [12] and George et al. [10].
7. Emerging Trends and Limitations: Recent advancements in laparoscopic-assisted cecostomy and minimally invasive techniques have improved outcomes, as noted by Bellamy et al. [23] and Vanek et al. [25]. However, limitations in resource availability and patient-specific factors, such as frailty and comorbidities, remain challenges to its widespread use [16,21].

### 4.1. Effectiveness and Outcomes of Cecostomy

The studies reviewed demonstrate that cecostomy is effective in providing rapid decompression for both malignant and functional colonic obstructions, with high success rates and relatively low perioperative morbidity. Perrier et al. [1] reported that cecostomy effectively alleviated malignant colonic obstructions, allowing for stabilization and reducing the need for emergent resections, which carry higher risks in critically ill patients. Similarly, Dalbaşı et al. [2] highlighted the use of cecostomy as a temporary measure in left-sided colonic obstructions, demonstrating its utility in bridging patients to definitive surgery while minimizing immediate complications.

The outcomes of cecostomy in the context of functional colonic obstructions, particularly Ogilvie’s syndrome, are particularly promising. Miller et al. [3] showed that early decompression via cecostomy in patients with pseudo-obstruction provided rapid symptom relief and reduced the need for emergent bowel resection, which is associated with higher morbidity and mortality. These findings suggest that cecostomy not only improves acute outcomes but also reduces the overall surgical burden for patients presenting with colonic obstruction.

Alwan-Walker et al. [5] illustrated the lifesaving potential of cecostomy in cases of acute functional obstruction secondary to conditions like neutropenic colitis, where immediate decompression is needed to prevent bowel perforation and sepsis. The lack of major complications in these reports highlights the safety and feasibility of cecostomy, particularly in high-risk patients where surgical resection may be contraindicated.

### 4.2. Comparisons with Other Decompressive Techniques

The decision to use cecostomy over other decompressive techniques, such as endoscopic stenting, colostomy, and ileostomy, is complex and depends on patient-specific factors, including the cause, location, and severity of the obstruction, as well as patient comorbidities and stability.

Endoscopic stenting is often preferred for its minimally invasive nature and immediate symptom relief, particularly in malignant obstructions. However, Tewari et al. [7] noted that stenting may not be feasible in all cases due to technical challenges, tumor characteristics, and risks of perforation or stent migration. In such scenarios, cecostomy remains a reliable alternative that provides effective decompression without the need for extensive surgery.

In contrast, colostomy and ileostomy offer definitive solutions by diverting the fecal stream away from the site of obstruction. These procedures, however, are associated with long-term stoma-related complications and impact on quality of life, which makes cecostomy a preferable option when a temporary measure is sufficient.

Cecostomy allows for reversible decompression, reducing the need for permanent diversion and offering a bridge to definitive surgery or further interventions when the patient is stabilized. This aspect of cecostomy makes it especially valuable in cases where the underlying obstruction is reversible or when the patient’s overall condition is expected to improve.

### 4.3. Limitations and Challenges in the Use of Cecostomy

Despite its benefits, the use of cecostomy is not without limitations. Risks such as infection, tube displacement, leakage, and bowel perforation pose potential challenges in both the short and long term. These complications, though relatively infrequent, require careful monitoring and appropriate postoperative management to ensure the cecostomy remains functional and does not contribute to further morbidity. Hoffmann and Jensen [4] reported a moderate rate of complications, including the need for additional interventions in some cases, underscoring the importance of patient selection and procedural expertise.

A significant challenge in cecostomy use is determining which patients are the best candidates for this procedure. While cecostomy is effective for distal colonic obstructions, its utility may be limited in cases of proximal obstruction, severe peritoneal contamination, or in patients with poor overall health status, where other interventions may be more appropriate. Given the nuances in decision-making, a multidisciplinary approach is often required to evaluate the patient’s condition and ensure that the selected intervention aligns with the clinical goals, whether they be curative, palliative, or temporary stabilization.

Additionally, the availability of resources and surgeon expertise play a role in the decision-making process. While open cecostomy can be performed in most surgical settings, the use of percutaneous cecostomy requires access to advanced imaging and technical skills, which may not be available in all healthcare environments. The lack of standardization in cecostomy techniques and management protocols further complicates its use, emphasizing the need for more research and clear guidelines to optimize its application.

### 4.4. Decline in Popularity of Cecostomy in the Era of Laparoscopic Surgery

Over the past decade, the landscape of surgical techniques has undergone a significant transformation, largely driven by advancements in minimally invasive procedures and innovations in technology. These developments have reshaped the way surgeons approach a variety of conditions, particularly those involving colonic obstructions and abdominal emergencies. Among these advances, laparoscopic surgery has emerged as a game-changer, fundamentally altering traditional practices and revolutionizing patient outcomes. This paradigm shift has also led to a marked decline in the use of traditional surgical techniques, including cecostomy, for several compelling reasons.

Firstly, laparoscopic techniques offer a significantly less invasive alternative to open surgery, with notable benefits for patients. These include reduced postoperative pain, lower risk of surgical site infections, shorter hospital stays, and faster recovery times. In addition, laparoscopic surgery provides improved cosmetic outcomes due to smaller incisions, which can enhance patient satisfaction and quality of life. Procedures such as laparoscopic resection and anastomosis, as well as laparoscopic colonic decompression, have become standard in many emergency settings, gradually replacing cecostomy as the preferred intervention. These approaches not only address the underlying cause of the obstruction but also reduce the morbidity associated with traditional open techniques, which typically involve larger incisions, longer recovery periods, and a higher risk of complications.

Secondly, the widespread adoption of endoscopic stenting as a bridge to surgery has further diminished the role of cecostomy, particularly in the management of malignant colonic obstructions. Endoscopic stenting offers the advantage of immediate decompression of the obstructed colon, allowing time for patients to be stabilized and optimized for definitive surgical intervention. By restoring bowel patency without the need for an external stoma, this minimally invasive technique provides both clinical and psychological benefits for patients. As a result, stenting has become a preferred option in many cases of malignant obstruction, reducing the reliance on more invasive and less convenient alternatives such as cecostomy.

Moreover, the advent of laparoscopic techniques has enabled more precise and targeted interventions in cases of functional colonic obstructions, such as Ogilvie’s syndrome. This condition, characterized by acute colonic distension without a mechanical blockage, has traditionally been managed with cecostomy when conservative measures fail. However, laparoscopic-assisted cecostomy and other minimally invasive decompressive measures have been shown to achieve comparable outcomes with fewer complications. The ability to visualize and address the distended colon with precision, while minimizing trauma to surrounding tissues, has further established laparoscopic approaches as a superior alternative in such cases.

Finally, advancements in surgical training, instrumentation, and imaging technology have facilitated the development of alternative decompressive techniques that are both more effective and more patient-friendly. For example, the integration of real-time imaging modalities, such as fluoroscopy and ultrasound, has improved the safety and accuracy of minimally invasive procedures. These advancements have enabled surgeons to expand the scope of laparoscopic and endoscopic techniques, making them viable options in scenarios that previously required open surgery or cecostomy. While cecostomy remains a valuable procedure in certain emergency scenarios—such as when minimally invasive options are contraindicated, unavailable, or technically unfeasible—it has become increasingly limited to these specific contexts.

The cumulative effect of these innovations has led to a significant shift in the surgical management of colonic obstructions. As the popularity of cecostomy declines, there is a growing need for further research to directly compare its long-term outcomes and cost-effectiveness with those of laparoscopic and endoscopic approaches. Such studies should investigate not only clinical outcomes but also patient-reported measures, such as quality of life and satisfaction with treatment. These data will be critical in refining clinical guidelines, optimizing patient care, and ensuring that each technique is applied in the most appropriate clinical context. By embracing these advancements while acknowledging the enduring value of cecostomy in select cases, the surgical community can continue to improve outcomes for patients with colonic obstructions.

### 4.5. Future Directions and Clinical Implications

The literature reviewed emphasizes the importance of individualized treatment planning for patients with colonic obstructions, where cecostomy can be tailored to meet specific clinical needs.

Further research is needed to refine patient selection criteria, assess the long-term outcomes of cecostomy versus alternative decompressive techniques, and develop standardized protocols for postoperative care and follow-up. Studies comparing cecostomy to newer minimally invasive interventions could provide valuable insights into its relative efficacy and help guide treatment decisions in emergency settings.

Moreover, understanding the quality of life impact and the cost-effectiveness of cecostomy compared to other decompressive techniques is essential for developing patient-centered care approaches. Given that cecostomy is often used as a temporary measure or as a bridge to definitive surgery, evaluating the timing of intervention, optimal duration of decompression, and appropriate timing for stoma reversal or closure will help improve overall outcomes and reduce healthcare burdens.

## 5. Conclusions

Cecostomy remains a valuable tool in the emergency management of colonic obstructions, providing effective decompression, symptomatic relief, and an opportunity for further surgical planning.

While it has proven benefits over certain decompressive techniques, its use must be balanced against the patient’s clinical context, the potential risks, and the availability of alternative interventions.

Given its versatility as both a temporary and definitive measure, cecostomy plays a critical role in bridging patients to further treatment, improving their stability, and preventing life-threatening complications.

Further comparative research is needed to optimize the selection criteria for cecostomy in emergency surgical settings, ensuring that this valuable procedure continues to provide strong clinical outcomes in appropriately selected cases.

## Figures and Tables

**Table 1:** The studies analyzed in this review.

Author and Year	Study Design	Population Studied	Type of Colonic Obstruction	Indications for Cecostomy	Outcomes and Effectiveness	Complications Reported
Perrier et al. (2020) [1]	Retrospective Cohort	113 patients with malignant colonic obstruction	Malignant colonic obstruction	Acute obstruction unsuitable for immediate resection	High success rate of decompression, reduced perioperative morbidity	Tube displacement (5%), local infection (3%)
Dalbași et al. (2023) [2]	Case Series	50 patients with left colonic carcinoma	Left-sided colonic carcinoma with obstruction	Temporary decompression as a bridge to definitive surgery	Effective in relieving symptoms, facilitating stabilization for later resection	No major complications reported
Miller et al. (2017) [3]	Prospective Study	30 patients with colonic pseudo-obstruction (Ogilvie’s syndrome)	Functional obstruction (Ogilvie’s syndrome)	Failure of conservative management, need for rapid decompression	Rapid relief of symptoms, prevention of emergency bowel resection	Leakage around tube site (10%)
Hoffmann and Jensen (1984) [4]	Case Review	60 patients with various causes of obstruction	Malignant and benign obstructions	Need for immediate decompression due to risk of perforation	Effective decompression, improved outcomes in unstable patients	Infection (8%), need for additional intervention (15%)
Alwan-Walker et al. (2014) [5]	Case Report	1 patient with neutropenic colitis	Functional obstruction secondary to neutropenic colitis	Lifesaving decompression	Successful relief of stabilization of patient	None reported
Yeo and Lee (2019) [6]	Review Article	N/A—Literature Review	Various colonic emergencies	Analysis of decompressive techniques in emergency settings	Review of management techniques, emphasizing urgency and efficacy	N/A—Review Article
Tewari et al. (2015) [7]	Prospective Analysis	Adults with malignant large bowel obstruction	Malignant large bowel obstruction	Safety and efficacy of percutaneous cecostomy for decompression	Demonstrated safety and feasibility in high-risk patients	Minor infections, no major complications
Khayyat et al. (2022) [8]	Systematic Review	Adults with malignant bowel obstruction	Malignant large bowel obstruction	Alternative to stenting in technically challenging cases	Favorable outcomes in critical patients	Leakage (4%), rare tube obstruction
Vanek et al. (2023) [9]	Case Series	Patients with Ogilvie′s syndrome	Functional pseudo-obstruction	Management of Ogilvie′s syndrome when conservative therapy fails	High efficacy in decompressing distended colon	None reported
George et al. (2021) [10]	Retrospective Cohort	75 patients with acute obstructions	Malignant and benign obstructions	Bridge to definitive resection or palliative decompression	Improved survival rates in palliative cases	Stoma site infection (7%)
Mori et al. (2018) [11]	Retrospective Study	90 patients with left-sided carcinoma	Left-sided colonic carcinoma	Stabilization before definitive resection	Reduction in mortality rates in emergency surgery	Tube-related infections (5%)
Singh et al. (2016) [12]	Prospective Cohort	Patients undergoing emergency decompression	Various obstructive pathologies	Emergency stabilization and symptomatic relief	Significant decrease in operative time	Minor wound infection (3%)
Zhang et al. (2020) [13]	Retrospective Study	60 patients with colonic obstructions	Malignant and benign obstructions	Emergency decompression in critically ill patients	High efficacy as a bridge to surgery	Rare tube displacement
Lee et al. (2021) [14]	Prospective Cohort	80 patients with obstructive carcinoma	Malignant colonic obstruction	Temporary decompression to delay major surgery	Success in avoiding immediate colectomy	Minimal complications
Kumar et al. (2023) [15]	Retrospective Study	45 elderly patients with colonic obstructions	Functional and malignant obstructions	Management of frail patients unsuitable for surgery	High symptom relief, improved quality of life	Local skin irritation (8%)
Jones et al. (2019) [16]	Systematic Review	Studies on emergency colonic decompression	Malignant and benign obstructions	Comparison of decompressive methods	Favorable for bridging and symptom relief	General risks reported
Ivanov et al. (2020) [17]	Case Series	35 patients with functional obstruction	Ogilvie’s syndrome	Failed conservative therapy	Immediate decompression, reduced hospital stays	None reported
Nakamura et al. (2021) [18]	Prospective Analysis	Pediatric patients with bowel obstruction	Functional and mechanical obstruction	Relief of obstruction in critical pediatric cases	Effective decompression, minimal recurrence rates	None Reported
Ahmed et al. (2017) [19]	Retrospective Cohort	Patients with advanced malignancy	Malignant obstructions	Palliative decompression to improve symptoms	High palliative success rates	Rare leakage (2%)
Patel et al. (2022) [20]	Systematic Review	Malignant obstruction management	Malignant bowel obstruction	Alternatives to surgery in high-risk patients	Safe and effective in reducing emergency surgeries	Infection (6%)
Murphy et al. (2019) [21]	Case Series	Geriatric patients with obstructions	Malignant and functional obstructions	Emergency decompression in frail elderly patients	High symptom relief and stabilization	Tube displacement (3%)
Castillo et al. (2020) [22]	Retrospective Study	50 patients with complex obstructions	Advanced malignancy	Bridge to palliative chemotherapy or definitive resection	High patient satisfaction, reduced morbidity	None reported
Bellamy et al. (2018) [23]	Review Article	Literature on functional obstruction	Functional pseudo-obstruction	Review of decompressive measures in non-mechanical obstructions	Highlights benefits of cecostomy as a safe measure	N/A– Review Article
Novak et al. (2022) [24]	Prospective Cohort	60 patients with colorectal emergencies	Malignant obstructions	Temporary decompression for stabilization	High survival rates with reduced emergency complications	Leakage (5%)

## Data Availability

All data generated or analyzed during this study were derived from publicly available sources and published peer-reviewed articles. No new datasets were created, and all data can be accessed from the respective publications cited in the references.
